# A new vision of definition, commentary, and understanding in clinical and translational medicine

**DOI:** 10.1186/2001-1326-1-5

**Published:** 2012-05-03

**Authors:** Xiangdong Wang

**Affiliations:** 1 Department of Respiratory Medicine, Center of Biomedical Research Center, Shanghai Respiratory Research Institute, Shanghai Key Laboratory of Organ Dysfunction, Fudan University Zhongshan Hospital, Shanghai, China

## Abstract

There is growing evidence to the importance of translational science and medicine in the improvement of patient outcome, even though the definitions of translational science, translational medicine, and clinical and translational medicine need to be further clarified. In the present perspective, we collected commentaries and descriptions about clinical and translational medicine from some members of Clinical and Translational Medicine editorial board to stimulate the discussion and help the understanding better.

## 

There is growing evidence to the importance of translational science and medicine in the improvement of patient outcome, even though the definitions of translational science, translational medicine, and clinical and translational medicine need to be further clarified. Clinical and translational medicine is expected to include scientific and regulatory investigations to translate preclinical researches to clinical application with a specific emphasis on new biotechnologies, biomaterials, bioengineering, disease-specific biomarkers, cellular and molecular medicine, omics science, bioinformatics, applied immunology, molecular imaging, drug discovery and development, and regulation and health policy. It is believed that clinical and translational medicine will benefit and improve novel diagnostics/prognostics and therapeutics for clinical use, post-genomic knowledge and experience, and/or new disciplines that reflect additional levels of complexity. We should clarify the bioethics at the interface and paradigms between technology and society, academies and industries, as well as publics and private models. Translational medicine should meet the demands of maintaining or expanding the biomedical workforce and education programs that attract and retain young people in the translational and biomedical sciences. In the present perspective, we collected commentaries and descriptions about clinical and translational medicine from some members of Clinical and Translational Medicine editorial board to stimulate the discussion and help the understanding better.

**Barry S. Coller** (David Rockefeller Professor of Medicine; Head, Allen and Frances Adler Laboratory of Blood and Vascular Biology; Physician-in Chief of the Rockefeller University Hospital; and Vice President for Medical Affairs, Rockefeller University) defines translational science as, “The application of the scientific method to address a health need.” In contrast to basic investigation, which has the generation of new knowledge as its primary goal, the primary of goal of translational science is improvement in human health.

**John Hutton** (Professor of Pediatrics, Vice President and Director, Biomedical Informatics, Cincinnati Children's Hospital Medical Center, University of Cincinnati) describes that a perfectly reasonable, “official” definition of translational research should be “Translational research transforms scientific discoveries arising from laboratory, clinical or population studies into new clinical tools and applications that improve human health by reducing disease incidence, morbidity and mortality” modified from “Transforming Translation – Harnessing Discovery for Patient and Public Benefit” (Report of the Translational Research Working Group of the National Cancer Advisory Board, US NIH, 2007).

**Dr György Marko-Varga** is the Head of Div. Clinical Protein Science & Imaging, Dept. of Measurement, Technology and Industrial Electrical Engineering, Lund University, Sweden, and Professor, 1st Department of Surgery, Tokyo Medical University, Japan. He was the lead principle scientist of the AstraZeneca Global Proteomics Network and developed clinical biomarker platforms for clinical studies. He also serves as the president of Swedish Proteomics Society and vice-president of European Proteomics Association. Dr Marko-Varga emphasizes that the modern health care is currently and globally undergoing a big revolution, where innovative therapies and novel technology advancements are having profound impacts on improving patient care and managing costs. Certainly, the latest frontiers in micro and nano-technology which allows high resolution protein sequence separation as well as Mass Spectrometry imaging that can resolve drug deposition that are localized in tumor environments.

One cornerstone within Health Care that has had great impact on cancer patients is the increasing number of personalized medicines being introduced into the market. The interest in personalized medicines actually results from understanding the molecular biology of diseases. This is mandatory, where the alignment of molecular signatures with particular disease forms, and our ability to measure markers associated with prognosis and outcomes is at the focus of Clinical and Translational Medicine. In this respect, personalized medicines treatment has now become a part of large cohort studies. I have been involved in large such non-small cell lung cancer (NSCLC) in several studies in Asia, and the outcome and impact has been overwhelming. In my view, the Clinical and Translational Medicine is a research field that bridges knowledge of disease processes, gained by in vitro and experimental animal models, with the disease pathways found in humans.

It is my vision that we expect from the research field of Clinical and Translational Medicine that a final goal is to develop helps the suffering patients with new diagnostics, drug products, and new medical knowledge for treating disease throughout the phases of clinical development. As an example, the Japanese authorities are working on a concept that will require for new drugs introduced to the market, that a diagnostic test is available improve on drug efficacy. The Biomarker assay is associated to the drug, and the patient stratification. We are expecting that such drug products will prove to be more efficacious within the targeted disease process, but also free from unwanted side effects and toxicity.

**William Rhodes** (Senior Vice President, Corporation Strategy and Development of BD, from a Life Sciences Professional’s View on CTM) defines that remarkable advances in technologies and the resulting methods used to explore and unlock the biology of cells are now being employed to discover, elucidate and create well-defined and strategically selected treatment modalities for numerous diseases. These same tools may then use to diagnose and classify the disease, select patients for whom the therapy is best suited, and then employed to monitor treatment effectiveness.

A confluence of technologies and capabilities has occurred making this possible – powerful methods for probing cells, such as mass spectrometry, flow cytometry, gene sequencing, image analysis, various detection modalities for measuring cause and effect within, on and between cells – all are directed towards not only understanding the cells in question, but also the diseases they cause, or in some cases, combat.

With the advent of such powerful tools has come a deluge of data – which now, because of significant advances in computational capability, information technology, data text mining and the like, can be effectively reviewed, analyzed and discoveries made.

Clinical translational medicine has emerged – based on the synthesis of information gleaned from multiple investigative sources, human biology and diseases are better understood and therapies more rapidly identified and tested, which, taken together, result in improved patient treatment and outcomes.

**Dr Dongming Hou** (Senior Scientist in Saint Joseph’s Hospital Atlanta/Saint Joseph’s Translational Research Institute, and Clinical Research Associate Professor at Department of Pharmacy Practice of Mercer University) thinks that translating scientific discoveries from a potential new medical device, drug, method, molecule into modern therapies also need a multidisciplinary team, which include scientist, physician, biomedical engineers, quality control, regulatory, business etc. Safety may be more important requirement for preclinical than efficacy especially in device field. Since human is much more complex than animal, the efficacy in preclinical model sometimes is not translate equally.

**Stephen I. Hsu** (one of global leading scientists in translational medicine) emphasized that the topic of a "social mission" should be also important for clinical translational scientists, to think ahead about how to translate in a manner that will enable public health models for disease prevention or treatment in low-resource settings to be realistically and successfully implemented. High technology approaches can either produce expensive therapies with relatively small impact if the target population is small (gene therapy or stem cell & regenerative medicine for rare diseases, etc.), or it can produce affordable low-tech and self-administered therapies that are targeted towards addressing common diseases (e.g. pandemics such as obesity and its related complications such as type 2 diabetes, highly prevalent cancers prevented by novel low-cost screening and treatment) and are specifically developed to be effective and available to both the most wealthy and the most impoverished. Medical advancement should be informed by the need to achieve global health equity. A social mission is part of our mandate as “translationists”. And often it starts by conceptualizing the public health care model that is most likely to work, identifying the challenges that must be overcome and then developing and bringing to market novel diagnostics, prognostics and therapeutics that meet urgent and unmet global needs.

**Claudio Spada** (Consultant of Information Systems and Communication Technology, Anaesthesia and Intensive Care Department, Fatebenefratelli & Ophthalmiatric Hospital, Milan, Italy), suggested Clinical and Translational Medicine as “The art to use scientific concepts and procedures to create interactions and synergies between different scientific medical disciplines and clinical practices, with a multi-modal process that integrates the exchange of information in order to optimize the efficiency and efficacy of the research, and advance the knowledge that can improve the patient care with better clinical applications”. Given the present scenario of the scientific publishing market and the specificity of the publication, the journal of CTM should also be seen as hub or platform for training programs in translational research, considering also the potentiality and opportunity of the on-line systems and technology. CTM should also propose and promote initiatives that use the new-generation interactive educational tools, like open Internet platform, to fulfill the unmet need of disseminating new knowledge in clinical and translational medicine that can lead to a better clinical practice. Some references related to these ideas include Patay BA, Topol EJ. The unmet need of education in genomic medicine. Am J Med. 2012 Jan; 125(1):5–6; Rubio DM, Schoenbaum EE, Lee LS, Schteingart DE, Marantz PR, Anderson KE, Platt LD, Baez A, Esposito K. Defining translational research: implications for training. Acad Med. 2010 Mar;85(3):470–5; Woo KT, Wong KS, Lee EJ, Chan CM. How can we Improve Clinical Research in Clinical Practice with Better Research Outcome? Ann Acad Med Singapore. 2011 Nov;40(11):499–8.

In conclusion, it is important and critical to understand the definition and concept of clinical and translational medicine and allow the variations of the understanding. The present Commentary is expected to initiate and stimulate the discussion of understanding about clinical and translational medicine.

